# A CozE Homolog Contributes to Cell Size Homeostasis of Streptococcus pneumoniae

**DOI:** 10.1128/mBio.02461-20

**Published:** 2020-10-27

**Authors:** Gro Anita Stamsås, Marine Restelli, Adrien Ducret, Céline Freton, Pierre Simon Garcia, Leiv Sigve Håvarstein, Daniel Straume, Christophe Grangeasse, Morten Kjos

**Affiliations:** a Faculty of Chemistry, Biotechnology and Food Science, Norwegian University of Life Sciences, Ås, Norway; b Molecular Microbiology and Structural Biochemistry, CNRS, Université de Lyon, Lyon, France; Carnegie Mellon University

**Keywords:** cell division, morphogenesis, penicillin-binding proteins, peptidoglycan

## Abstract

Control of peptidoglycan assembly is critical to maintain bacterial cell size and morphology. Penicillin-binding proteins (PBPs) are crucial enzymes for the polymerization of the glycan strand and/or their cross-linking via peptide branches. Over the last few years, it has become clear that PBP activity and localization can be regulated by specific cognate regulators. The first regulator of PBP activity in Gram-positive bacteria was discovered in the human pathogen Streptococcus pneumoniae. This regulator, named CozE, controls the activity of the bifunctional PBP1a to promote cell elongation and achieve a proper cell morphology. In this work, we studied a previously undescribed CozE homolog in the pneumococcus, which we named CozEb. This protein displays the same membrane organization as CozE but is much more widely conserved among *Streptococcaceae* genomes. Interestingly, *cozEb* deletion results in cells that are smaller than their wild-type counterparts, which is the opposite effect of *cozE* deletion. Furthermore, double deletion of *cozE* and *cozEb* results in poor viability and exacerbated cell shape defects. Coimmunoprecipitation further showed that CozEb is part of the same complex as CozE and PBP1a. However, although we confirmed that CozE is required for septal localization of PBP1a, the absence of CozEb has no effect on PBP1a localization. Nevertheless, we found that the overexpression of CozEb can compensate for the absence of CozE in all our assays. Altogether, our results show that the interplay between PBP1a and the cell size regulators CozE and CozEb is required for the maintenance of pneumococcal cell size and shape.

## Abstract (precis)

Penicillin-binding proteins (PBPs), the proteins catalyzing the last steps of peptidoglycan assembly, are critical for bacteria to maintain cell size, shape, and integrity. PBPs are consequently attractive targets for antibiotics. Resistance to antibiotics in Streptococcus pneumoniae (the pneumococcus) are often associated with mutations in the PBPs. In this work, we describe a new protein, CozEb, controlling the cell size of pneumococcus. CozEb is a highly conserved integral membrane protein that works together with other proteins to regulate PBPs and peptidoglycan synthesis. Deciphering the intricate mechanisms by which the pneumococcus controls peptidoglycan assembly might allow the design of innovative anti-infective strategies, for example, by resensitizing resistant strains to PBP-targeting antibiotics.

## INTRODUCTION

Streptococcus pneumoniae (pneumococcus) is a Gram-positive bacterium usually found as a commensal in the nasopharynx of healthy adults and children. It does, however, have the potential to become pathogenic and is a frequent cause of community-acquired diseases (1). S. pneumoniae is associated with a variety of infections that can range in severity from otitis media to pneumonia or meningitis (2). As a result of the increased proportion of pneumococcal strains resistant to several antibiotics, the pneumococcus is part of the WHO list of priority pathogens for research and development of new antibiotics (3).

S. pneumoniae has also emerged over the last 10 years as an important model organism to study bacterial cell division and morphogenesis (4, 5). The pneumococcus displays an ovoid cell shape, reminiscent of a rugby ball, that divides by binary fission along successive parallel planes that are perpendicular to the long axis of the cell. One striking feature of the pneumococcus is that it assembles the cell wall and, notably, the peptidoglycan (PG) mesh, which confers the cell shape and guarantees the physical integrity of the cell, only from the midcell region. Indeed, the pneumococcus is devoid of the actin-like protein MreB, which drives lateral insertion of PG in many other bacteria (4). Rather, PG assembly is organized only by the tubulin-like protein FtsZ, which forms a treadmilling ring (Z ring) at midcell and organizes the cell division machinery (divisome) (6). PG assembly should therefore be finely balanced to serve both in cell elongation (peripheral assembly) and in building of the septal cross-wall separating the two daughter cells (septal assembly). A major regulator of the pneumococcal PG assembly is the serine-threonine-kinase StkP, which regulates the function of several proteins in the divisome and other enzymes required for PG polymerization and/or remodeling (7–9). StkP notably phosphorylates the cell division proteins DivIVA, MapZ, MacP, and EloR and regulates PBP2x and the glucosaminidase LytB through direct interaction (7, 9–16).

The central precursor of the PG mesh, which consists of a lipid-linked *N*-acetylglucosamine (NAG)–*N*-acetylmuramic acid (NAM)–pentapeptide named lipid II, is synthetized on the inner side of the plasma membrane and then exported across the membrane by the flippase MurJ (17). Then, the glycosyltransferase activity of two types of PG synthases, the class A PBPs (penicillin-binding proteins) and the SEDS (shape, elongation, division and sporulation) proteins, polymerizes the glycan strand, while the transpeptidase activity of class A and B PBPs joins the peptide branches, resulting in the PG mesh. There is an intricate interplay between these different enzymes: two SEDS proteins, RodA and FtsW, are dedicated to the polymerization of glycan strands during cell elongation and cross wall synthesis, respectively (18–20). Recently, it was shown that dedicated class B PBPs form a complex with specific partner SEDS proteins to cross-link glycan strands and build the primary structure of the PG mesh (19). In the pneumococcus, the two class B PBPs of the pneumococcus, PBP2b and PBP2x, work together with RodA and FtsW in peripheral and septal PG assembly, respectively (20). In contrast, the role of class A PBPs is less clear, and notably, their numbers vary from one species to the other. It was recently shown, however, that class A PBPs can work independently of the SEDS–class B PBP complexes and contribute to the remodeling of the PG mesh required during expansion and/or after cell wall damages (21, 22). The class A PBPs PBP1a, PBP1b, and PBP2a encoded by the pneumococcus are not essential, and individual deletion leads to weak morphological and growth defects. However, some of the combined deletions are either synthetic lethal or detrimental for cell division (23).

With the characterization of LpoA and LpoB in Escherichia coli, evidence has been provided that the activity of class A PBPs is controlled by specific regulators (24, 25). The same is true in the pneumococcus, in which two proteins, CozE and MacP, that are unrelated to LpoA and LpoB interact with and regulate the function of PBP1a and PBP2a, respectively (10, 26, 27). CozE and MacP are not homologous but are suggested to perform similar functions, as the CozE-PBP1a and MacP-PBP2a pairs are required for proper pneumococcal cell division and morphogenesis (10, 26). While MacP is mainly restricted to streptococci, CozE is more widespread and distributed in many bacterial clades. In S. pneumoniae, CozE was found to control PBP1a by directing its activity to midcell (26, 28). A recent study demonstrated that CozE proteins are also required for normal cell division in Staphylococcus aureus (29). Importantly, two CozE homologs were detected in S. aureus, named CozEa and CozEb. While CozEa and CozEb could be deleted individually without major growth or morphological defects, the Δ*cozEa* Δ*cozEb* double mutation was lethal (29). Together with knockdowns showing abnormal morphologies, these results suggest that CozEa and CozEb possess overlapping functions in cell division (29).

Here, we report that S. pneumoniae also encodes a second CozE homolog (Spr1357 in strain R6, SPD_1332 in strain D39). For clarity and consistency with the S. aureus study and our phylogenetic analysis, this protein is termed CozEb and the original CozE (Spr0777 in strain R6, SPD_0768 in strain D39) is referred to as CozEa in this work. Our experiments demonstrated that the deletion of the two genes generates opposing effects on cell size. In addition, we found that the two proteins are part of the same molecular complex as PBP1a. Although only expression of *cozEa* is needed to allow the localization of PBP1a at the division septum, we found that overexpression of *cozEb* compensates for the absence of CozEa and restores proper cell shape and growth as well as the septal PBP1a localization. These findings identify a new morphogenic protein and demonstrate that the protein triad comprising CozEa, CozEb, and PBP1a is required for normal pneumococcal cell shape. They further illustrate the complexity of the network of protein interactions involved in the bacterial cell morphogenesis.

## RESULTS

### S. pneumoniae encodes two CozE homologs.

**FIG 1:**
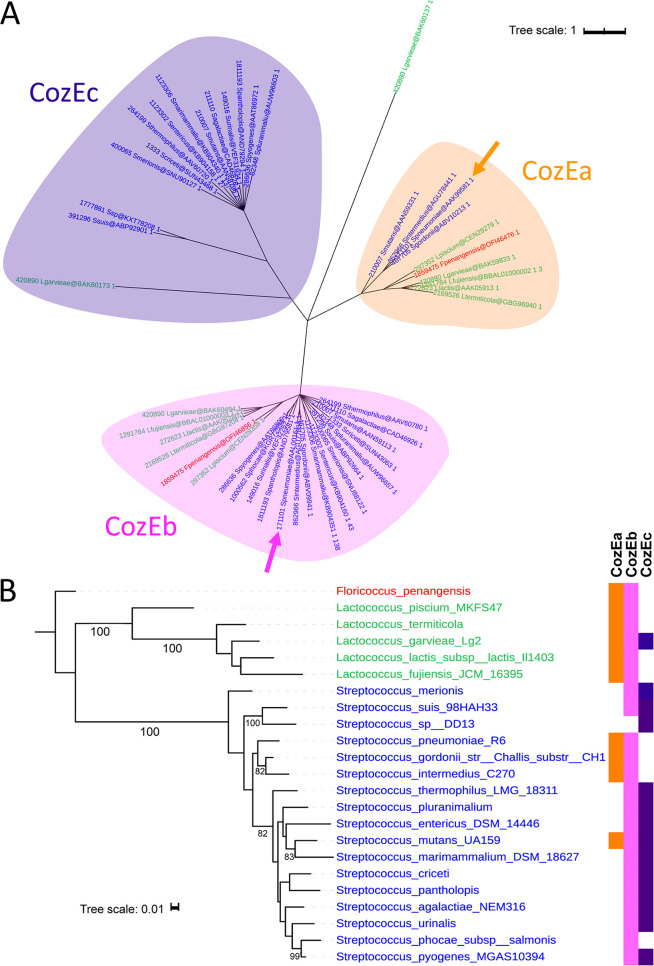
Phylogenomic analysis of CozE in *Streptococcaceae*. (A) Phylogenetic tree of the CozE protein family. The colors of leaves indicate genus (red, *Floricoccus*; green, *Lactococcus*; blue, *Streptococcus*). The groups, named CozEa, CozEb, and CozEc, are distinguished by different colors. The sequences of Streptococcus pneumoniae R6 are indicated by pink and orange arrows. The branches with a support of <80% have been collapsed. The bar represents the average number of substitutions per site. (B) Taxonomic distribution of CozEa, CozEb, and CozEc in *Streptococcaceae*. The tree was inferred using the RpoB marker (see Materials and Methods). The colors of leaves indicate genus (red, *Floricoccus*; green, *Lactococcus*; blue, *Streptococcus*). The presence of a copy of a protein is indicated by a colored square. The supports of >80% are indicated at the branches. The bar represents the average number of substitutions per site.

Homology searches using the pneumococcal CozE (Spr0777; here referred to as CozEa) (26) as the query showed that S. pneumoniae encodes a second protein (Spr1357) belonging to the same family (Pfam PF01594) (28). Spr1357 displays 28% identity and 54% similarity with CozEa (Fig. S1A). In addition, the two proteins have similar predicted membrane topologies (Fig. S1B and C), confirming that Spr1357, here named CozEb, is a CozEa homolog. To determine the conservation of CozE proteins within the *Streptococcaceae*, we performed a phylogenetic analysis of proteins derived from 23 representative *Streptococcaceae* genomes (Fig. 1A). Interestingly, the analysis revealed that all genomes encode two CozE homologs. However, three separate subgroups of CozE proteins have emerged within *Streptococcaceae* (Fig. 1A). The two pneumococcal homologs, CozEa and CozEb, belong to two different subgroups, the subgroup of CozEb being present in almost all *Streptococcaceae* (Fig. 1B). In contrast, the distribution of CozEa and that of the third subgroup, here named CozEc, is patchier and limited to some species only (Fig. 1B). Except for Streptococcus mutans and Lactococcus garvieae which harbor proteins from all three CozE subgroups, CozEa is never found together with CozEc in *Streptococcaceae* genomes. Consequently, a member of the CozEb subgroup is conserved in most, or possibly almost all, *Streptococcaceae* genomes, suggesting that members of this subgroup may be of particular importance. The function of CozEb in *Streptococcaceae* has not been studied to date, and we therefore set out to understand the function of this protein using S. pneumoniae as a model organism.

10.1128/mBio.02461-20.1 FIG S1 (A) Sequence alignment of Spr0777 (CozEa) and Spr1357 (CozEb). The alignment was made using Clustal Omega (http://www.ebi.ac.uk/Tools/msa/clustalo/) and constructed using ESPript 3 (http://espript.ibcp.fr/). Predicted secondary structures (helices) are indicated above the alignment, and identical and similar residues are highlighted in the alignment. Transmembrane helices are highlighted in yellow. The large extracellular loop predicted in both CozEa and CozEb as well as the shorter loop specific of CozEb is indicated in green. (B and C) Transmembrane topology predictions of CozEa (B) and CozEb (C) were made using Protter (http://wlab.ethz.ch/protter/). N and C termini are indicated. Predicted transmembrane spans are numbered from 1 to 8. Download FIG S1, PDF file, 1.6 MB. Copyright © 2020 Stamsås et al. 2020 Stamsås et al. This content is distributed under the terms of the Creative Commons Attribution 4.0 International license.

### *cozEb* is not essential for growth but is important for proper cell size control.

**FIG 2:**
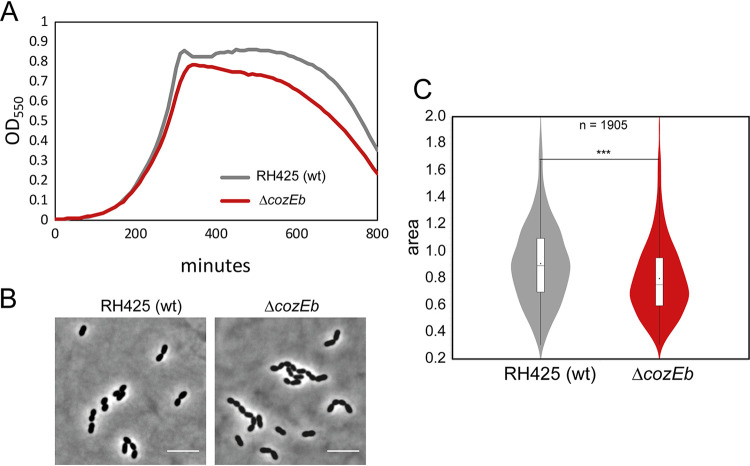
Deletion of *cozEb* results in cells with reduced size. (A) Growth curves of RH425 (wild type [wt]; generation time, 49 min) and the Δ*cozEb* mutant (GS1250; generation time, 53 min). (B) Representative phase-contrast images of RH425 (wt) and GS1250 (Δ*cozEb*). Bars, 5 μm. (C) Violin plot of the cell areas of RH425 (wt) and Δ*cozEb* (GS1250) as determined using MicrobeJ (30). The box indicates the 25th to the 75th percentile, and the whiskers indicate the minimum and the maximum values. The mean and the median are indicated with a dot and a line in the box, respectively. The two-tailed *P* value (***, *P* < 0.0001) was derived from a Mann-Whitney test. A total of 1,905 cells were analyzed.

**FIG 3:**
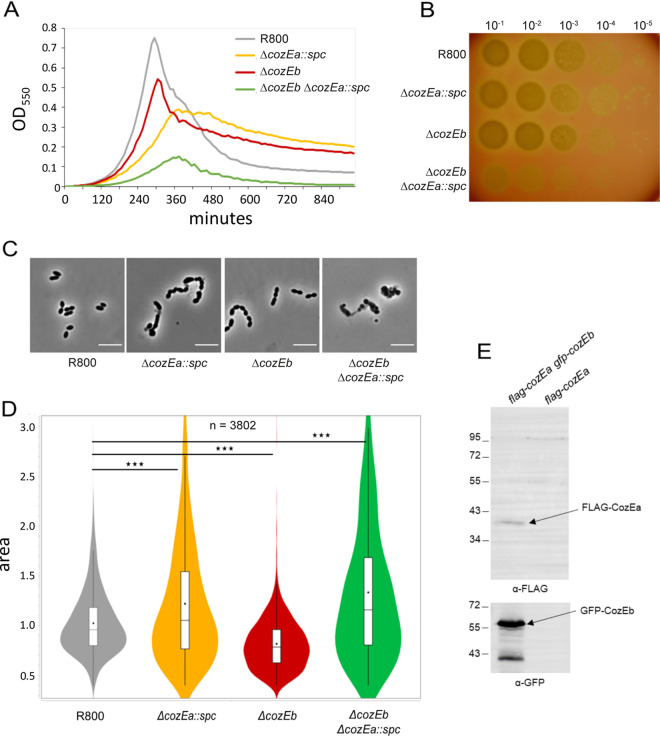
*cozEa* and *cozEb* have a synthetic link. (A) Growth curves of R800 (generation time, 37 min), the *cozEa*::*spc* strain (generation time, 61 min), the Δ*cozEb* strain (generation time, 49 min), and the double-deletion strain *cozEa*::*spc* Δ*cozEb*. (B) Viability assay of the same strains. Cells were grown to an OD_550_ of 0.1, diluted, and spotted on THY (Todd-Hewitt broth supplemented with yeast extract) agar plates supplemented with 3% (vol/vol) horse blood and incubated overnight at 37°C. (C) Representative micrographs of the R800, *cozEa*::*spc*, Δ*cozEb*, and *cozEa*::*spc* Δ*cozEb* strains. Bars, 5 μm. (D) Cell size analysis of the same strains. In the violin plots, the boxes indicate the 25th to the 75th percentile and the whiskers indicate the minimum and the maximum values. The mean and the median are indicated with a dot and a line in the box, respectively. The two-tailed *P* value (***, *P* < 0.0001) was derived from a Mann-Whitney test. A total of 3,802 cells were analyzed. (E) Coimmunoprecipitation of Flag-tagged CozEa with GFP-CozEb. A GFP trap was used to bind GFP-CozEb, and the immunoprecipitate was analyzed by Western blotting using a Flag antibody and a GFP antibody. The Flag-CozEa strain is shown as a control.

To study the function of CozEb in S. pneumoniae, we first constructed a markerless deletion of the gene in the R6 derivative strain RH425. *cozEb* was deleted with normal transformation efficiency. The growth of the RH425Δ*cozEb* mutant was only slightly reduced compared to that of the wild-type (strain RH425) in C+Y medium at 37°C (Fig. 2A). Notably, however, cell chaining increased significantly, since the fraction of cells that were in chains (≥4 cells) was 58% for the Δ*cozEb* strain (*n* = 443 cells) compared to only 4% for the RH425 wild-type (*n* = 382 cells) (Fig. 2B). Furthermore, by performing automated cell size analysis using MicrobeJ (30), we found that the Δ*cozEb* cells were smaller than wild-type cells (Fig. 2B). Reduced cell size was observed also for the set of nonchaining Δ*cozEb* cells (Fig. S2A). In a similar fashion, the deletion of *cozEb* in another related R6 derivative S. pneumoniae strain, R800, led to the same phenotype: 17% of the R800Δ*cozEb* cells were in chains (≥4 cells; *n* = 1,094), as opposed to 0% of the wild-type cells (*n* = 1,016), and both the length and width were reduced for the R800Δ*cozEb* strain compared to the wild type (Fig. 3A to D and Fig. S2B). This observation differs from what was reported for the R6 strains devoid of *cozEa*. CozEa-depleted cells also formed long chains of cells but with variable morphologies, and cell rounding and swelling were frequently observed (26, 28). We therefore obtained the *cozEa*::*spc* deletion construct used previously (26) and were able to confirm the phenotypic differences between the two deletion mutant constructs (R800Δ*cozEb* versus R800Δ*cozEa*::*spc*) (Fig. 3A to D; Fig. S2C). The analysis showed that in contrast to Δ*cozEb* cells, which are uniformly smaller than wild-type cells, R800Δ*cozEa*::*spc* cells have highly variable cell morphologies. While some have reduced lengths and widths, there is also a large fraction of Δ*cozEa*::*spc* cells that are strikingly larger than wild-type cells (Fig. 3D). When only nonchaining cells were analyzed, the same trend was detected. Indeed, the fractions of both very small and very large cells are higher for Δ*cozEa*::*spc* cells (19% of Δ*cozEa*::*spc* cells have an area of <0.6 μm^2^ compared to 7% for wild-type and 23% for Δ*cozEb* cells, while 20% of Δ*cozEa*::*spc* cells are >1.2 μm^2^ compared to 15% for wild-type and 1.8% for Δ*cozEb* cells). Deletions of *cozEa* and *cozEb* thus seem to have different and even opposing effects on the pneumococcal cell morphology and size.

10.1128/mBio.02461-20.2 FIG S2 (A) Violin plots of the cell areas of RH425 (wt) and nonchaining Δ*cozEb* cells (GS1250) as determined using MicrobeJ. Δ*cozEb* cells in chains were removed from the analysis. The box indicates the 25th to the 75th percentile, and the whiskers indicate the minimum and the maximum values. The mean and the median are indicated with a dot and a line in the box, respectively. The two-tailed *P* value (***, *P* < 0.0001) was derived from a Mann-Whitney test. The numbers of cells analyzed were 1,290 (for RH425) and 256 (for Δ*cozEb* cells). (C and D) Plots of cell lengths versus cell widths for (B) R800 (*n* = 992; grey dots) and the Δ*cozEb* strain (*n* = 1,084; red dots) and (C) R800 and the Δ*cozEa*::*spc* strain (*n* =1,108; yellow dots). Download FIG S2, TIF file, 1.3 MB. Copyright © 2020 Stamsås et al. 2020 Stamsås et al. This content is distributed under the terms of the Creative Commons Attribution 4.0 International license.

The original CozE, CozEa, was also reported as being essential in S. pneumoniae D39 but not in S. pneumoniae R6 (26). This difference was ascribed to different alleles of *pbp1a* (D39 versus R6; T124A and D388E). Therefore, we also tested the deletion of *cozEb* in strain D39 (31). A *cozEb* deletion mutant was readily obtained, and analysis of cell size parameters also showed a reduction of the average cell size (Fig. S3A and B). Together, these results show that Δ*cozEb* mutants display a cell size defect distinct from that of *cozEa* deletion mutants, suggesting that CozEb is involved together with CozEa in pneumococcal cell size homeostasis and that the two homologs may have a complementary function.

10.1128/mBio.02461-20.3 FIG S3 (A) Micrographs and (B) cell area analyses of D39V (wt) and GS1336 (Δ*cozEb*::Janus). Bars, 5 μm. The box plot indicates the 25th to the 75th percentile, and the whiskers indicate the minimum and the maximum values. The mean and the median are indicated with a dot and a line in the box, respectively. The two-tailed *P* value (***, *P* < 0.0001) was derived from a Mann-Whitney test. A total of 2,229 cells was analyzed. Download FIG S3, TIF file, 1.4 MB. Copyright © 2020 Stamsås et al. 2020 Stamsås et al. This content is distributed under the terms of the Creative Commons Attribution 4.0 International license.

### Synthetic relationship between *cozEb* and *cozEa*.

To study the functional relationship between CozEa and CozEb, we looked at the growth and the cell morphology of the respective single- and double-deletion mutants. As observed above and previously (26, 28), the cell size and the growth of the single deletion mutants were both affected (Fig. 2 and 3A to D; Fig. S2), with more severe effects for the deletion of CozEa. Interestingly, the double-deletion mutant was even more affected: cells grew very poorly (Fig. 3A) and were hardly viable (Fig. 3B), and cell morphologies were severely perturbed, with the presence of numerous lysed cells (Fig. 3C and D). Similar perturbed morphologies were also observed for the double deletion in the RH425 genetic background (Fig. S4). This suggests that there is a synthetic link between these two genes in S. pneumoniae, as the cells hardly tolerate the absence of both. To further confirm this link, we sought to determine whether the two proteins were able to interact *in vivo*. For that, we constructed a strain expressing both CozEa fused to a Flag-tag and CozEb fused to green fluorescent protein (GFP), and we performed coimmunoprecipitation experiments. Upon GFP trapping, a Flag signal was detected in the eluted fraction, indicating that the two proteins copurified (Fig. 3E) and strongly suggesting that they work together in the same protein complex.

10.1128/mBio.02461-20.4 FIG S4 (A) Representative micrographs RH425 and GS1375 (*cozEa*::*spc* Δ*cozEb*). Bar, 5 μm. (B) Cell size is severely perturbed in strain GS1375 (*cozEa*::*spc* Δ*cozEb*) compared to wild-type strain RH425. The violin plots indicate the 25th to the 75th percentile, and the whiskers indicate the minimum and the maximum values. The mean and the median are indicated with a dot and a line in the box, respectively. In total, 1,378 cells were analyzed. Download FIG S4, TIF file, 2.0 MB. Copyright © 2020 Stamsås et al. 2020 Stamsås et al. This content is distributed under the terms of the Creative Commons Attribution 4.0 International license.

**FIG 4:**
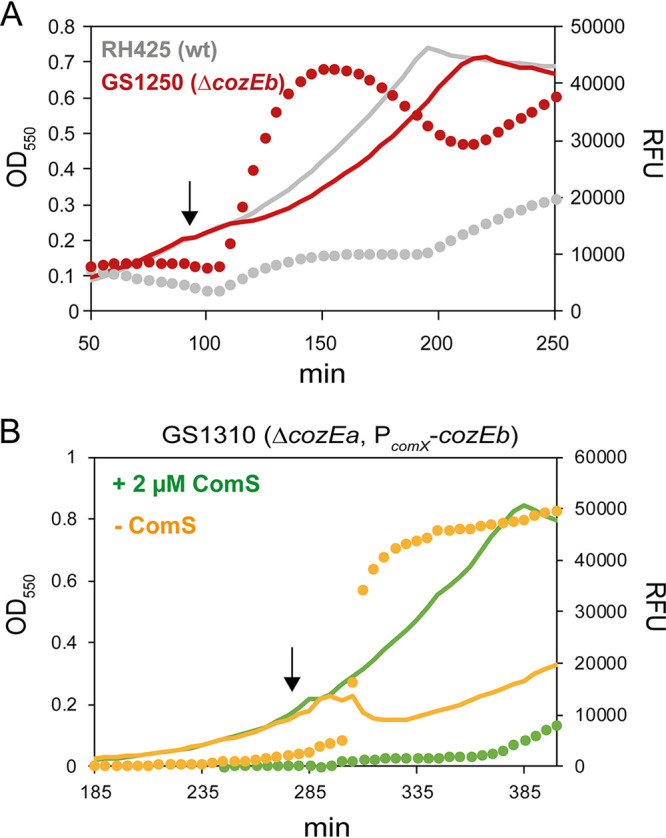
DNA release assay demonstrating CbpD sensitivity during competence. Growth curves (solid lines; OD_550_) and DNA release (dotted lines; relative fluorescence units [RFU]) were measured in real time. Competence was induced at the time points indicated with arrows by addition of CSP-1 (250 ng/ml). DNA release was measured as described before (28) by growing cells in the presence of Sytox green nucleic acid stain, which fluoresces upon DNA binding. Results are expressed as RFU. An increase in RFU after addition of CSP indicates sensitivity to CbpD. See Materials and Methods for details. (A) DNA release assay of wild-type RH425 and GS1250 (Δ*cozEb*). (B) DNA release assay of strain GS1310 (Δ*cozEa* P*_comX_*-*cozEb*) to demonstrate that overexpression of *cozEb* can complement the CbpD-hypersensitive phenotype of the Δ*cozEa* strain. Cells were pregrown in medium with 2 μM ComS, before inoculation in medium with 2 μM ComS (green) or without ComS (orange). CSP-1 was added when cultures reached an OD_550_ of 0.2 (arrows).

To further investigate the functional link between CozEa and CozEb, we investigated the sensitivity of *ΔcozEb* cells to the hydrolytic activity of the cell wall hydrolase CbpD (32, 33). S. pneumoniae secretes the cell wall hydrolase CbpD upon competence induction (32) and is able to protect itself by expressing an immunity protein ComM (34). Depletion of some proteins involved in cell wall synthesis, including CozEa, results in hypersensitivity to CbpD upon competence induction (28). Upon analysis of the Δ*cozEb* strain (GS1250), we observed that this strain becomes sensitive to CbpD upon competence induction, as observed by a drop in optical density at 550 nm (OD_550_) and increased release of DNA as measured by Sytox green binding (Fig. 4A). It should be noted that lysis in the Δ*cozEb* mutant is clearly lower than previously observed for a *cozEa* depletion strain (28), again showing that the lack of CozEa results in a more severe phenotype than the lack of CozEb. Altogether, these findings suggest that CozEa and CozEb are functionally linked and that both are important for building a fully intact pneumococcal cell wall.

### Overexpression of *cozEb* can complement the lack of *cozEa*.

**FIG 5:**
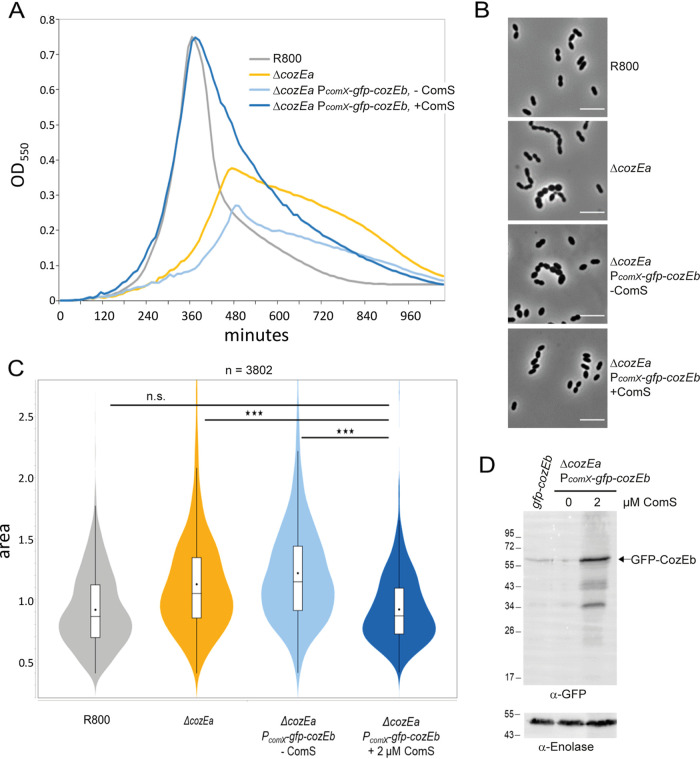
Overexpression of *cozEb* can rescue the growth and cell size defect of the *cozEa* deletion in S. pneumoniae R800 (A) Growth curves of R800 wild-type cells, the Δ*cozEa* mutant, and the complementation strain (Δ*cozEa P_comX_-gfp-cozEb*) grown with or without the inducer peptide ComS (2 μM). (B) Representative phase-contrast images of the same strains. Bars, 5 μm. (C) Cell area analysis of the same strains. In the violin plots, the boxes indicate the 25th to the 75th percentile and the whiskers indicate the minimum and the maximum values. The mean and the median are indicated with a dot and a line in the box, respectively. The two-tailed *P* value (***, *P* < 0.0001) was derived from a Mann-Whitney test. A total of 5,510 cells were analyzed. (D) Western blot using anti-GFP antibody to show that GFP-CozEb is overexpressed upon induction with the inducer peptide ComS (2 μM) compared to wild-type and noninduced conditions.

As the results described above show that *cozEa* and *cozEb* are functionally linked, we wanted to study whether elevated *cozEb* expression could complement the Δ*cozEa* deletion. For that, we first made a markerless deletion of *cozEa* in strain R800 using the Janus system. We checked that this strain displayed a growth defect and cell shape aberrations similar to those of the *cozEa*::*spc* strain analyzed before (Fig. 3 and 5). Then, a copy of *cozEb* fused to *gfp* was ectopically expressed from the P*_comX_* promoter inserted at an ectopic locus in the *cozEa* deletion background. Expression from the P*_comX_* promoter can be induced by addition of the extracellular inducer peptide ComS (35). Growing this strain in the absence of ComS (mimicking a *cozEa* deletion) resulted in severe growth and morphology defects as previously shown (Fig. 3 and 5) (26). However, upon induction of the P*_comX_* promoter by addition of 2 μM ComS, GFP-CozEb was efficiently overexpressed (Fig. 5D) and the growth and cell morphology defects of Δ*cozEa* were suppressed (Fig. 5A to C). This was confirmed upon expression of untagged CozEb under the same conditions (Fig. S5). Also, as mentioned above, depletion of CozEa results in a CbpD-hypersensitive phenotype upon competence induction. We show here that overexpression of CozEb could restore the CbpD-resistant phenotype (Fig. 4B). Together, these results show that overexpression of CozEb can compensate for the lack of CozEa in S. pneumoniae.

10.1128/mBio.02461-20.5 FIG S5 Overexpression of *cozEb* can rescue the growth defect of the *cozEa* deletion. Strain GS1310 (RH425 Δ*cozEa* P*_comX_*-*cozEb*) was pregrown in medium with ComS and then diluted in medium with 2 μM ComS or without ComS. (A) Growth curves and (B) phase-contrast micrographs. Cells were imaged at an OD_600_ of 0.1 to 0.2. Bars, 5 μm. Download FIG S5, TIF file, 2.7 MB. Copyright © 2020 Stamsås et al. 2020 Stamsås et al. This content is distributed under the terms of the Creative Commons Attribution 4.0 International license.

### CozEb is in complex with PBP1a but does not affect its localization.

**FIG 6:**
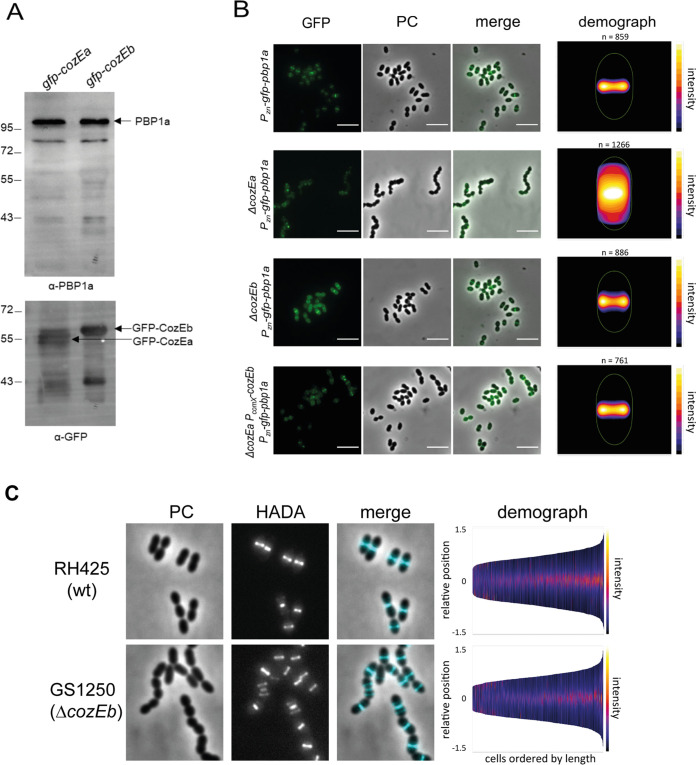
CozEb is in complex with PBP1a. (A) Coimmunoprecipitation experiments demonstrating that both CozEa and CozEb are in complex with PBP1a *in vivo*. Strains expressing *gfp-cozEa* or *gfp-cozEb* were subjected to GFP trapping, and the immunoprecipitated fraction was probed with an anti-PBP1a antibody. (B) Localization of GFP-PBP1a in the R800, Δ*cozEa*, and Δ*cozEb* strains and the Δ*cozEa* complementation strain overexpressing *cozEb* (Δ*cozEa P_comX_*-*cozEb*). Cells were grown in 0.15 mM ZnCl_2_ to induce *gfp-pbp1a* expression and induced with 2 μM ComS to allow overexpression of CozEb in the complementation strain (bottom). Bars, 5 μm. (C) Localization of PG synthesis in RH425 and the Δ*cozEb* strain as measured by staining with the fluorescent d-amino acid HADA. Cells were incubated with HADA for 90 s (see Materials and Methods). Phase-contrast, HADA signal, and merged images are shown. Bars, 5 μm. In the demographs, the cells are ordered by length and the HADA signal is indicated by a color scale. The numbers of cells analyzed for RH425 and GS1250 were 1,225 and 680, respectively.

As mentioned above, CozEa was previously suggested to direct cell elongation in S. pneumoniae by interacting with PBP1a (26). Therefore, we also performed coimmunoprecipitation assays with strains expressing either GFP-labeled CozEa or GFP-labeled CozEb. Blots were then probed with specific anti-PBP1a antibodies. Our analysis confirmed the interaction between CozEa and PBP1a (Fig. 6A). Interestingly, coimmunoprecipitation also showed an interaction between CozEb and PBP1a (Fig. 6A). Thus, CozEb seems to be part of the same complex as CozEa and PBP1a.

PBP1a localizes to midcell in S. pneumoniae (36, 37). In line with what was reported before (26), we showed that removal of *cozEa* results in complete loss of GFP-PBP1a localization (Fig. 6B). Surprisingly, deletion of *cozEb* had no effect on GFP-PBP1a localization (Fig. 6B). Indeed, PBP1a demonstrates a clear midcell localization in the absence of CozEb. Consistently, the deletion of *cozEb* has no impact on the localization of the nascent peptidoglycan, which is still localized properly at midcell, as analyzed by staining with fluorescent d-amino acids (7-hydroxycoumarincarbonylamino-d-alanine [HADA]) (38) (Fig. 6C). Notably, however, overexpression of *cozEb* could fully restore the localization of PBP1a at midcell in the Δ*cozEa* genetic background (Fig. 6B). These experiments demonstrate that although CozEb is likely not directly responsible for controlling the localization of PBP1a and nascent-peptidoglycan incorporation, its overexpression renders CozEa dispensable, suggesting an intricate interplay between the triad CozEa, CozEb, and PBP1a.

## DISCUSSION

Bifunctional class A PBPs in S. pneumoniae were recently shown to have unique roles, independent of the monofunctional class B PBPs, in S. pneumoniae (21). The exact biological function of class A PBPs during cell wall synthesis remains to be determined, but recent results indicate that their main role is repair, remodeling, or strengthening of PG rather than primary PG synthesis (21, 22, 39). Consequently, the activity of these PBPs needs strict control in time and space to maintain the cell wall homeostasis ensuring the cell shape and integrity during the division process. Two regulators have been shown to control the activity of class A PBP in the pneumococcus. The membrane protein CozE, referred to as CozEa in this study, is required for the localization of PBP1a at the division septum where PG is produced (26, 27). On the other hand, MacP, a protein unrelated to CozEa, is a phosphorylation-dependent activator of PBP2a (10). In this work, we report that CozEb is a homolog of CozEa and part of the same protein complex together with PBP1a. It was shown previously that CozEa also interacts with MreC and MreD to direct pneumococcal cell elongation (26). One can therefore hypothesize that CozEb also contributes to the same process. Supporting this, our results show that CozEb is involved in pneumococcal cell morphogenesis and/or cell size homeostasis, since deletion of *cozEb* resulted in cell chaining, reduced cell size, and increased susceptibility to the cell wall hydrolase CbpD. On the other hand, the deletion of *cozEa* generates cells with variable morphologies which are either bigger or smaller than wild-type cells. Furthermore, the CbpD hypersensitivity, which indicates reduced PBP1a activity resulting from the *cozEa* depletion (28), is more severe than observed here for Δ*cozEb*. It therefore seems that these two homologs have different and even opposing effects on pneumococcal cell morphology. A reasonable and interesting hypothesis would be that CozEa and CozEb have opposing effects on PBP1a and cell size expansion and that the coordinated action of both proteins would allow balanced PG maturation, giving rise to the ovoid cell shape of the pneumococcus. Our finding that the double deletion (*cozEa* and *cozEb*) resulted in a synthetic sick phenotype with poorly growing cells with highly aberrant morphologies, while both single-deletion mutants of *cozEa* and *cozEb* are viable, is in agreement with this hypothesis.

The synthetic sick phenotype observed with the double-deletion mutant is to some extent analogous to what was observed in S. aureus. In this bacterium, *cozEa* and *cozEb* single-deletion mutants were obtained, but a Δ*cozEa* Δ*cozEb* mutant could not be made (29). In S. aureus, CozEa and CozEb were also shown to interact, similar to what was found here. One can therefore infer that related regulatory processes involving CozEa and CozEb would control a cognate PBP in S. aureus as in the pneumococcus, although such interactions have not yet been identified. However, there are important differences between the results in S. aureus and S. pneumoniae. While neither of the single deletions in S. aureus produced any severe phenotype, we found here that in S. pneumoniae, the deletion phenotype was much more pronounced in the Δ*cozEa* strain than in the Δ*cozEb* strain. One might speculate that this is related to the differences in repertoire and roles of class A PBPs between these species. While S. pneumoniae encodes three nonessential class A PBPs (PBP1a, PBP1b, and PBP2a), S. aureus encodes only a single, essential class A PBP, namely, PBP2. Therefore, the uncontrolled action of PBP2 likely resulting from the absence of CozEa and CozEb could explain their synthetic lethality in S. aureus, since no other class A PBPs are encoded to compensate for PBP2 dysfunction. In contrast, the aberrant activity of PBP1a in a pneumococcal Δ*cozEa* Δ*cozEb* mutant can be compensated for by the two other class A PBPs. Interestingly, deletion of *pbp1a* and *pbp2a* is the unique synthetic lethal pair, while co-deletion of *pbp1a* and *pbp1b* or of *pbp1b* and *pbp2a* results only in some weak pneumococcal cell shape defects (23). Considering that PBP1a is misregulated in the Δ*cozEa* Δ*cozEb* mutant, it is tempting to speculate that PBP2a would be essential in these cells. Supporting this claim, we have never been able to delete *pbp2a* in such a strain. As mentioned previously, a recent report showed that PBP2a is regulated by the morphogenic protein MacP. In the same study, it is also demonstrated that MacP is able to interact directly with PBP1a. However, the functional relevance of this interaction has not been investigated. Consequently, a potential functional interconnection between the two CozE proteins and MacP cannot be excluded as well.

With CozE and MacP regulating PBP1a and PBP2a, respectively, PBP1b is the only class A PBP in S. pneumoniae for which no regulator has been found. It was consequently tempting to speculate that CozEb could be involved in the regulation of PBP1b. To address this, we performed coimmunoprecipitation experiments with the CozE proteins and PBP1b. Interestingly, we did observe a low-intensity PBP1b band, far less intense than the PBP1a band (Fig. 6A), coimmunoprecipitating with both CozEa and CozEb (Fig. S7). Even if robust conclusions cannot be drawn based on this single observation, the latter supports the hypothesis that also PBP1b may be part of the same complex as CozEa, CozEb, and PBP1a. Further investigations will be needed to assess the relevance of this potential interaction. In light of our findings, one can therefore suggest that an intricate regulatory mechanism, relying on a set of morphogenic proteins (including CozEa, CozEb, and MacP), would allow the coordination of class A PBPs in PG maturation and remodeling.

An interesting question emerging from our study concerns what differentiates CozEa and CozEb. Indeed, their deletions do not have the same impact on cell morphology, indicating that they should have intrinsic properties differentiating them. However, Δ*cozEa* cells are rescued from PBP1a delocalization, cell morphology defects, and CbpD hypersensitivity when *cozEb* is overexpressed from an ectopic promoter. Similar results have previously been obtained for the staphylococcal CozEa and CozEb proteins, whose overexpression in S. pneumoniae could complement the *cozEa* depletion phenotype (29). An interesting hypothesis is that CozEa and CozEb have some common properties but that one of the two possesses a specific feature dedicated to a precise function not performed by the other. In line with this hypothesis, we have found that overexpression of CozEa does not complement the absence of CozEb and cells are still smaller (Fig. S6). Considering this, CozEb would be the one harboring a special trait.

10.1128/mBio.02461-20.6 FIG S6 Overexpression of *cozEa* cannot complement the deletion of *cozEb.* The Δ*cozEb* P*_comX_*-*gfp*-*cozEa* strain was pregrown in medium with ComS and then diluted in medium with 2 μM ComS or without ComS. Cell were imaged at an OD_550_ of 0.1 to 0.2. (A) Representative micrographs of wild-type R800 and the complementation strain with or without ComS. (B) The violin plot indicates the 25th to the 75th percentile, and the whiskers indicate the minimum and the maximum values. The mean and the median are indicated with a dot and a line in the box, respectively. The two-tailed *P* value (***, *P* < 0.0001). was derived from a Mann-Whitney test. A total of 6,521 cells were analyzed. (B) Western blot using anti-GFP antibody to show that GFP-CozEa is overexpressed upon induction with 2 μM of the inducer peptide ComS, compared to wild-type and noninduced conditions. Download FIG S6, TIF file, 2.5 MB. Copyright © 2020 Stamsås et al. 2020 Stamsås et al. This content is distributed under the terms of the Creative Commons Attribution 4.0 International license.

10.1128/mBio.02461-20.7 FIG S7 Coimmunoprecipitation experiments of CozEa and CozEb with PBP1b. Strains expressing *gfp-cozEa* or *gfp-cozEb* were subjected to GFP trapping, and the immunoprecipitated fraction was probed with an anti-PBP1b antibody. Download FIG S7, TIF file, 1.0 MB. Copyright © 2020 Stamsås et al. 2020 Stamsås et al. This content is distributed under the terms of the Creative Commons Attribution 4.0 International license.

This hypothesis is in line with our phylogenomic studies showing that CozEb is the most conserved among *Streptococcaceae* genomes. Interestingly, the CozE family of proteins within *Streptococcaceae* is grouped into three subgroups; CozEa, CozEb, and CozEc. It is interesting that all *Streptococcaceae* genomes encode mainly two CozE proteins that belong to different subgroups. This suggests that their functions are dependent on each other but also that they fulfill different purposes. Sequence identity between proteins from different subgroups is always <30%, and comparative sequence analysis did not reveal any sequence features which were clearly conserved or variable between the three different subgroups. Our predictions of the secondary structures and membrane topologies of CozEa and CozEb, however, show some differences between the two proteins. Notably, the predicted length of the N-terminal end in the cytoplasm differs between CozEa and CozEb (Fig. S1B and C). With regard to loop conservation, there is a large extracellular loop connecting the transmembrane helices 3 and 4 and a shorter loop between helices 5 and 6 predicted in CozEb (Fig. S1). The large loop is also predicted in CozEa, although the sequence is poorly conserved (28% identity and 54% similarity between full-length CozEa and CozEb sequences but only 15% identity and 35% similarity between the predicted loops), while the latter loop is not even predicted in CozEa. These extracellular loops, which both are predicted to contain alpha-helices (Fig. S1A), could potentially be important for protein-protein interactions, and it will be particularly interesting to evaluate whether these predicted variations are responsible for the functional differences between CozEa and CozEb.

Numerous antibiotics currently used to treat bacterial infections target the enzymes involved in PG assembly, such as the PBPs. However, an increasing number of bacterial species are now resistant to these molecules. For the pneumococcus, resistance to β-lactam antibiotics is often associated with mutations in *pbp* genes (40, 41). For example, variants of *pbp2b* and *pbp2x* confer resistance to piperacillin and cefotaxin, respectively (42–44). In addition, it has been shown that defects associated with mutations conferring resistance, like in *pbp2b*, can be compensated for with mutations in other *pbp* genes, namely, *pbp1a* and *pbp2x* (45). Therefore, deciphering the intricacies of the underlying regulatory mechanism governed by these morphogenic proteins is likely to generate fundamental knowledge necessary to design innovative strategies for combating such antibiotic-resistant bacteria.

## MATERIALS AND METHODS

### Bioinformatics.

For homology searches, sequence alignments were performed using Clustal Omega (http://www.ebi.ac.uk/Tools/msa/clustalo/) and constructed using ESPript 3 (http://espript.ibcp.fr/). Topology predictions were made using Protter (http://wlab.ethz.ch/protter/). For phylogeny inference, proteomes of 23 representative *Streptococcaceae* were retrieved from NCBI (GenBank; ftp://ftp.ncbi.nlm.nih.gov/) to build a database. Homologs of CozE were identified using BLASTP v2.8.1+ (46). Starting from the sequence of Streptococcus pneumoniae CozEa (AAK99581.1) using an E-value of 10^−4^ as the threshold. The homologs were aligned using MAFFT v7.419 (L-INS-I option) (47). After manual curation, the alignment was trimmed using BGME v1.1 with matrix substitution BLOSUM30 (48) and used to infer a maximum-likelihood tree using IQ-TREE v1.6.10 (49). The best-suited evolutionary model was selected by applying the BIC criteria implemented in IQ-TREE (LG+F+R4). The groups were defined by the topology and the taxonomic distribution. The robustness of branches was assessed by 1,000 replicates of ultrafast bootstraps.

The marker RpoB was used to build the reference tree of *Streptococcaceae*. More precisely, RpoB sequences were identified by BLASTP v2.8.1+, starting from S. pneumoniae R6 sequence using an E-value of 10^−4^ as threshold. The homologs were aligned using MAFFT v7.419 (L-INS-I option). The alignment was used to infer a maximum-likelihood tree using IQ-TREE v1.6.10 (49). The best-suited evolutionary model was selected by applying the BIC criteria implemented in IQ-TREE (LG+R4). Figures were generated using iTOL (50). The robustness of branches was assessed by 1,000 replicates of ultrafast bootstraps.

### Bacterial strains, growth conditions, and transformation.

All strains used in this study are listed in Table S1. S. pneumoniae was routinely grown in C+Y medium (51) at 37°C without shaking. Transformation was done with standard protocols, by addition of 250 ng/ml CSP-1. When appropriate, S. pneumoniae was grown in either 250 μg/ml or 400 μg/ml of kanamycin, 200 μg/ml streptomycin, or 200 μg/ml spectinomycin.

10.1128/mBio.02461-20.8 TABLE S1 Plasmids and strains used in this study. Download Table S1, PDF file, 0.2 MB. Copyright © 2020 Stamsås et al. 2020 Stamsås et al. This content is distributed under the terms of the Creative Commons Attribution 4.0 International license.

### Strain construction.

All strains and plasmids are listed in Table S1 and all primers in Table S2. The primers associated with construction of the different strains are indicated in Table S1, in addition to the detailed description below. All strains generated in this study are available upon request.

10.1128/mBio.02461-20.9 TABLE S2 List of oligonucleotides used in this study. Download Table S2, PDF file, 0.05 MB. Copyright © 2020 Stamsås et al. 2020 Stamsås et al. This content is distributed under the terms of the Creative Commons Attribution 4.0 International license.

### (a) General information about strain construction using the Janus cassette.

In the R800 and RH425 genetic backgrounds, the Janus cassette was used for genetic manipulation. In a streptomycin-resistant background (*rpsL1*), the introduction of a Janus cassette (containing a kanamycin resistance gene and the *rpsL* wild-type allele) results in cells becoming resistant to kanamycin and susceptible to streptomycin (52). This allows the removal of the Janus cassette in a second step, by selection for streptomycin resistance. In general, for deletion of a gene or introduction of a fragment using the Janus cassette, ∼1,000 bp upstream and ∼1,000 bp downstream of the gene of interest are first amplified by PCR. These fragments are then fused to the Janus cassette by overlap extension PCR, before transformation of the full fragment into S. pneumoniae by natural transformation. When applicable, the Janus cassettes were subsequently removed by transforming the strain with a fused fragment containing the up- and downstream sequences. Correct deletions were verified by PCR and sequencing.

### (b) Deletion of *cozEb*.

*cozEb* was deleted using the Janus cassette system. The *cozEb* upstream fragment was amplified using the primer pair KHB498-KHB499 or 2639-2642, and the downstream fragment was amplified with the primer pair KHB500-KHB501 or 2663-2640. These fragments were fused to the Janus cassette (amplified by the primer pair Kan484.F-RpsL41.R or 536-537) by the use of the outer flanking primers. The final fragment was then introduced into the appropriate strain by natural transformation to obtain the Δ*cozEb*::Janus deletion strain. For removal of the Janus cassette, the upstream fragment was amplified with the primer pair KHB498-GS722 or 2639-2665 and the downstream fragment with the primer pair GS723-KHB501 or 2640-2664. The fragments were fused and transformed into the Δ*cozEb*::Janus strain to remove the Janus cassette.

### (c) Deletion of *cozEa*.

*cozEa* was deleted by allelic replacement with a spectinomycin resistance cassette or by using the Janus cassette system. For the former, the fragment Δ*cozEa*::*spc* fused to the upstream and downstream regions of *cozEa* was amplified with the primers 2789-2790 from the genomic DNA of the strain D39 Δ*pbp1a*::*kan* Δ*cozEa*::*spc* (26). This fragment was introduced into the strain R800 and into the Δ*cozEb* mutant by natural transformation.

Deletion of *cozEa* using the Janus cassette was done as described previously (28), using the primer pair KHB482-KHB483 or 2789-2660 to amplify the *cozEa* upstream region and KHB484-KHB485 or 2659-2790 to amplify the *cozEa* downstream region. The Janus cassette was amplified with the primer pair Kan484.F-RpsL41.R or 536-537. The three fragments were then fused before being introduced into the appropriate strain, resulting in a *ΔcozEa*::Janus strain. For removal of the Janus cassette, the primer pair KHB482-GS473 or 2789-2662 was used to amplify the *cozEa* upstream region and GS474-KHB485 or 2661-2790 was used to amplify the *cozEa* downstream region. The fragments were fused and transformed into the Δ*cozEa*::Janus strain.

### (d) Construction of *gfp-cozEb* and *gfp-cozEa*.

The *gfp*-*cozEb* and *gfp*-*cozEa* constructs were made using the Janus cassette system. For construction of *gfp-cozEb*, the upstream region of *cozEb* was amplified using the primers 2639 and 2689, and the *cozEb* sequence with its downstream region was amplified with the primers 2688 and 2640. The primers 2688 and 2689 hybridize with the *gfp* gene, which was amplified using primers 1368 and 1568. This allowed us to obtain a complete *gfp-cozEb* fragment that was inserted in the Δ*cozEb*::Janus strain (Spn1496).

For the *gfp-cozEa* strain, *gfp*-*cozEa* was amplified from the plasmid pACYC *bgaA*′::*P_fucose_-gfp-cozE_tetM*::*bgaA*′ *bla*, (26) using the primers 2216 and 2818. The upstream region and the downstream region of *cozEa* were amplified with the primers pairs 2789-2691 and 2817-2790, respectively, and fused to the *gfp-cozEa* fragment to obtain a complete fragment that was inserted into the Δ*cozEa*::Janus strain (Spn1680).

### (e) Construction of *flag-cozEa*.

The upstream region of *cozEa* was amplified using the primers 2789 and 2996, and the *cozEa* sequence with its downstream region was amplified with the primers 2820 and 2790. The primers 2996 and 2820 both have a Flag sequence and hybridize with each other. The complete fragment was transformed into the *ΔcozEa*::Janus locus of strain Spn1922.

### (f) Construction of P*_comX_-cozEb* for ectopic expression of *cozEb* in RH425.

P*_comX_* and its upstream region was amplified with primer pair KHB31-KHB36 from strain SPH131 (35). The downstream region was amplified with primers KHB33 and KHB34 using the same template. The primers GS730 and GS731 were used to amplify *cozEb*. Both of these primers have overhangs, allowing fusion of these three fragments. This final product was introduced into strain SPH154 (35) to construct the P*_comX_-cozEb* fusion.

### (g) Construction of P*_comX_-gfp-cozEb* for ectopic expression of *gfp-cozEb* in R800.

The upstream region and the P*_comX_* were amplified with primers 1946 and 2081 using the strain Spn1010 (ΔIS*1167*::P*1-*P*_comR_-comR*, *cpsN-cpsO*::P*_comX_*-Janus) as a template, and the downstream region was amplified with primers 1943 and 2082, using the same template. The primers 2670 and 2746 were used to amplify *gfp-cozEb* from the strain Spn1528. These primers have sequences that hybridize with 2082 and 2081, respectively. This allows the fusion of the three fragments by PCR. The final product was introduced into strain Spn1010.

### (h) Construction of *gfp-pbp1a*.

The localization of PBP1a was performed using the Δ*bgaA*::P*_Zn_-gfp-pbp1a* construct previously described (36). In this strain, *gfp-pbp1a* is expressed ectopically under Zn^2+^ induction. The P_Zn_::*gfp-pbp1a* construct was transferred into the desired strain by transformation with genomic DNA that was extracted from the Δ*bgaA*::P_Zn_*-gfp-pbp1a* strain.

### Growth analysis.

Growth analysis was routinely performed in 96-well microtiter plates. Exponentially growing cultures were diluted to the desired optical density, and OD_550_ was measured every 5th or 10th minute using a Synergy H1 hybrid reader (BioTek) or a Jasco V-630-Bio spectrophotometer (Jasco).

### DNA release assay with Sytox green to monitor lysis.

DNA release assay to monitor cell lysis upon competence induction was performed essentially as described previously (28). Sytox green nucleic acid stain (Invitrogen) fluoresces upon binding to DNA, and since the dye cannot be internalized by pneumococcal cells, emission of fluorescence is a marker for cell lysis. Briefly, cells were grown in 96-well plates (black plates, clear bottom; Corning) in the presence of 2 μM Sytox green nucleic acid stain (Invitrogen). Growth (OD_550_) and fluorescence emitted (excitation and emission wavelengths, 485 and 528 nm) were measured every 5th minute using a Synergy H1 hybrid reader (BioTek). When necessary, the cultures were induced to competence at an OD_550_ of ∼0.2 by the addition of 250 ng/ml CSP-1 (competence-stimulating peptide 1).

### Microscopy.

For phase-contrast microscopy, cells were grown to an OD_550_ of 0.2 to 0.3. For GFP-PBP1a imaging, cells were grown to an OD_550_ of 0.1. Expression of *gfp*-*pbp1a* was induced with 0.15 mM ZnCl_2_ for 1 h. For the HADA-labeling experiments, cells at an OD_550_ of 0.1 were incubated in medium with 250 μg/ml HADA (53) for 90 s. Cells were then washed with cold phosphate-buffered saline (PBS) and imaged immediately.

Cells were imaged on a Zeiss AxioObserver microscope with ZEN Blue software (Zeiss) through a 100× PC objective or on a Nikon TiE microscope with NIS-Elements (Nikon) through a 100× 1.45 numerical aperture objective. Both microscopes were fitted with an ORCA‐Flash4.0 V2 digital CMOS camera (Hamamatsu Photonics) for image capturing. Images were analyzed using ImageJ (http://rsb.info.nih.gov/ij/) and the plugin MicrobeJ (30).

### Coimmunoprecipitation and immunoblot analysis.

Cultures were grown at 37°C in C+Y medium until an OD_550_ of 0.4 was reached. Cells were centrifuged at 5,000 × *g* for 10 min. The pellets were suspended in buffer A (0.1 M Tris-HCl [pH 7.5], 2 mM MgCl_2_, 1 M sucrose) containing 6 μg/ml of DNase I and RNase (Sigma) A and 0.2 μg/ml protease inhibitor (Roche Diagnostics), incubated at 30°C for 30 min, and centrifuged at 5,000 × *g* for 10 min. The pellets were resuspended in buffer A with 100 U/ml of mutanolysin (Sigma) and 8 mg/ml of lysozyme. The cells were incubated for 15 min at room temperature. After centrifugation (10 min at 5,000 × *g*), the pellets were suspended in buffer B (0.1 M Tris-HCl [pH 7.5], 100 mM NaCl, 1 mM EDTA, 1 mM dithiothreitol [DTT], 5% [mass/vol] digitonin [Sigma], 8 mU/μl mutanolysin, 8 mg/ml lysozyme, 0.2 mg/ml protease inhibitor, and 6 μg/ml DNase/RNase). The lysate was incubated at 37°C for 30 min and centrifuged at 15,000 × *g* for 30 min. The supernatant was incubated for 2 h at 4°C with GFP trap slurry (Chromotek). The lysate was centrifuged at 2,700 × *g* at 2 min, and the pellet was washed three times with buffer C (10 mM Tris-HCl [pH 7,5], 0.5 mM EDTA, 150 mM NaCl, 0.05% digitonin [mass/vol] and 0.2 mg/ml protease inhibitor) and were eluted in 2× Laemmli loading buffer at 95°C for 10 min. The proteins were analyzed by SDS-PAGE and immunoblotting using an anti-PBP1a or anti-PBP1b antibody (37) at 1/20,000 and an anti-Flag antibody at 1/1,000 (Sigma). Goat anti-rabbit immunoglobulin horseradish peroxidase-conjugated secondary antibody (Bio-Rad) for the anti-PBP1a antibody and goat anti-mouse immunoglobulin horseradish peroxidase-conjugated secondary antibody (Bio-Rad) for the anti-Flag antibody were used at 1/5,000 to reveal the immunoblots.

For the immunoblot analysis, S. pneumoniae cells were resuspended in TE buffer (25 mM Tris-HCl [pH 7.5], 1 mM EDTA, 0.2 mg/ml protease inhibitor) and lysed by sonication. A 25-μg portion of crude extract was loaded onto an SDS-polyacrylamide gel for electrophoresis and electrotransferred on a polyvinylidene difluoride membrane. The membrane was incubated with an anti-GFP antibody at 1/10,000 (AMS Biotechnology) or anti-enolase antibody 1/50,000 (7). Goat anti-rabbit immunoglobulin horseradish peroxidase-conjugated secondary antibody (Bio-Rad) were used at 1/5,000 to reveal the immunoblots.
