# Supplementary figures and images for: A CozE Homolog Contributes to Cell Size Homeostasis of Streptococcus pneumoniae
Source: mBio. 2020 Oct 27;11(5):e02461-20. doi: 10.1128/mBio.02461-20 (PMC7593971; doi:10.1128/mBio.02461-20)

A

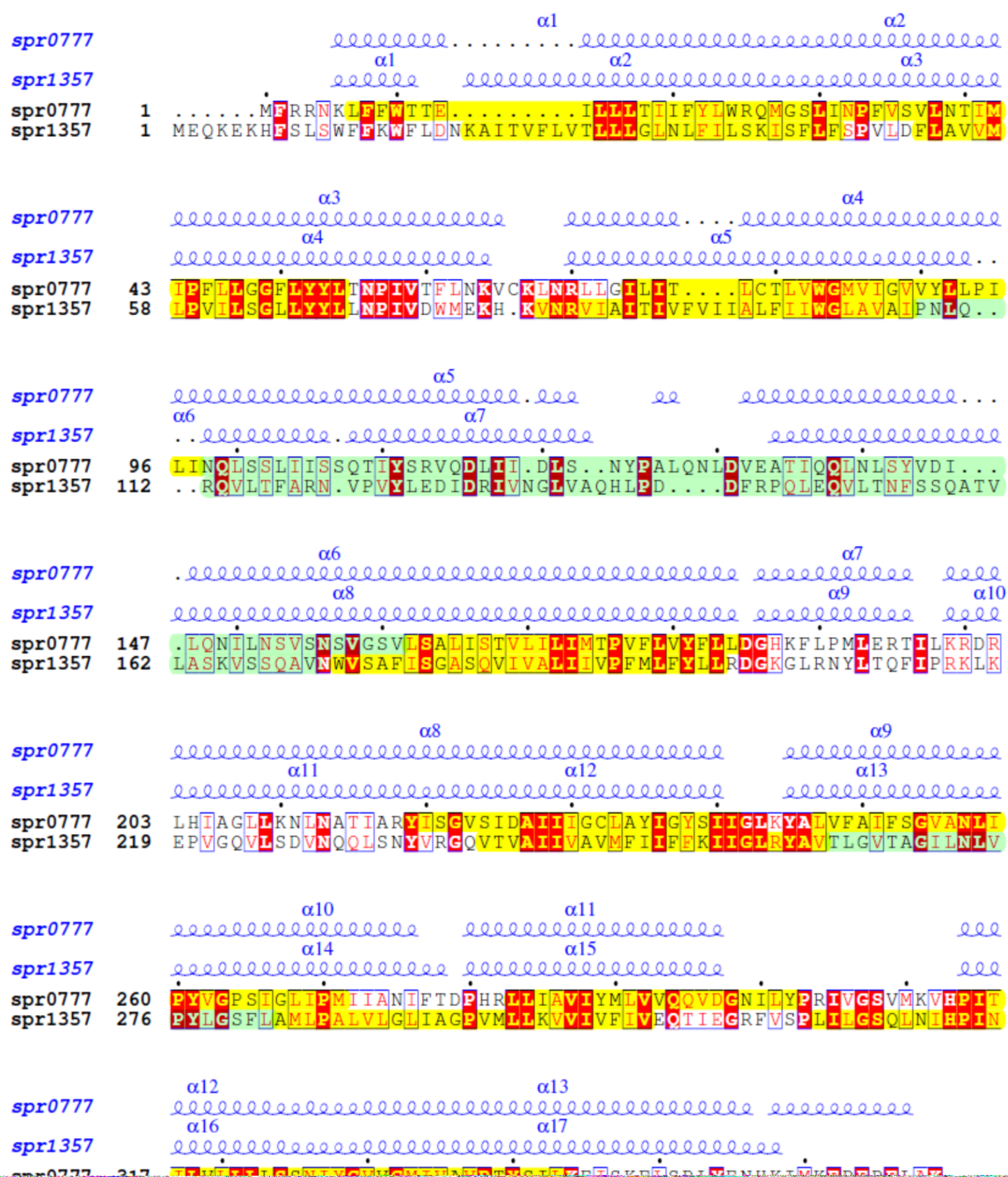

Supplement: FIG S1 [file mBio.02461-20-sf001.pdf]

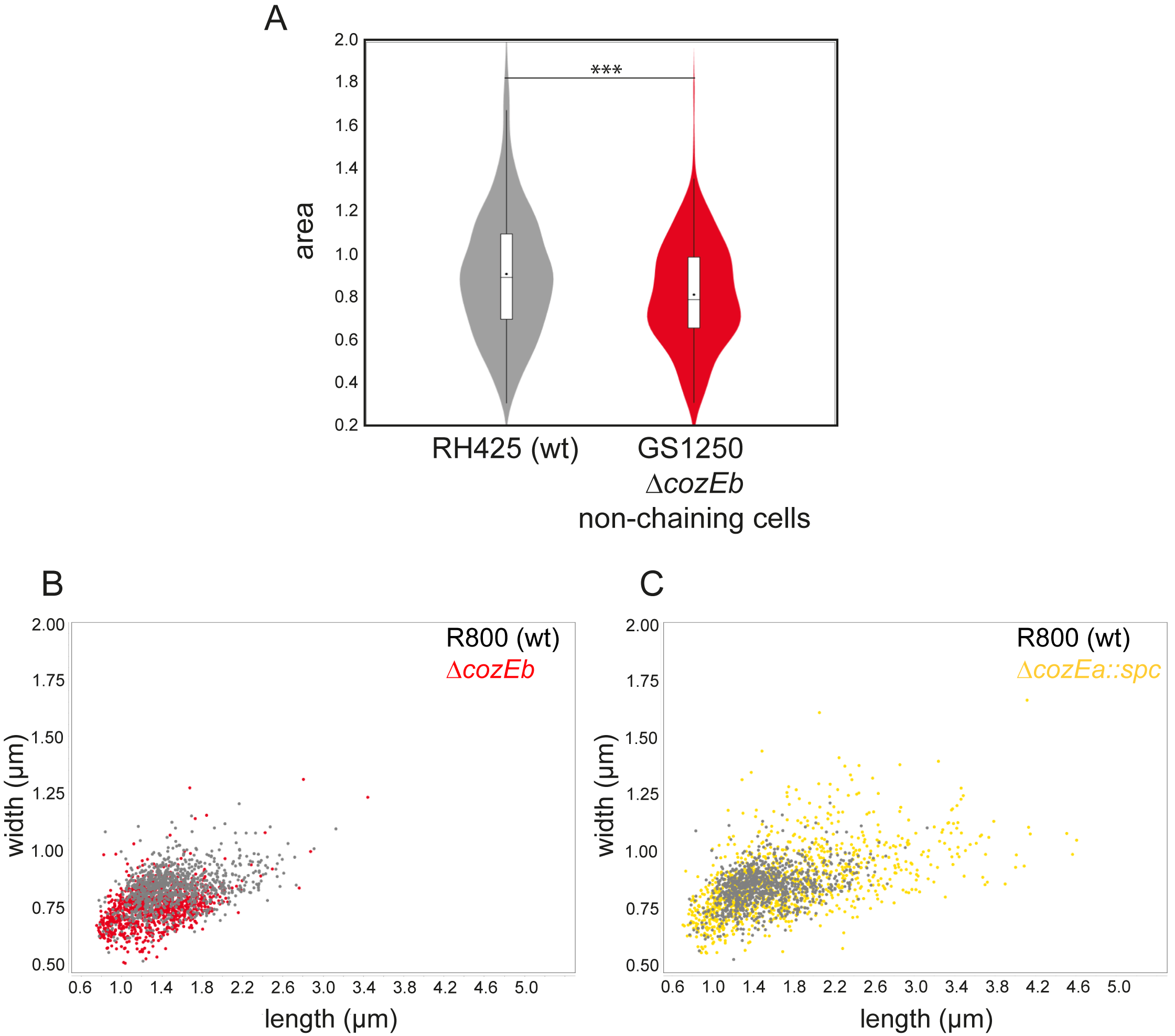

Supplement: FIG S2 [file mBio.02461-20-sf002.tif]

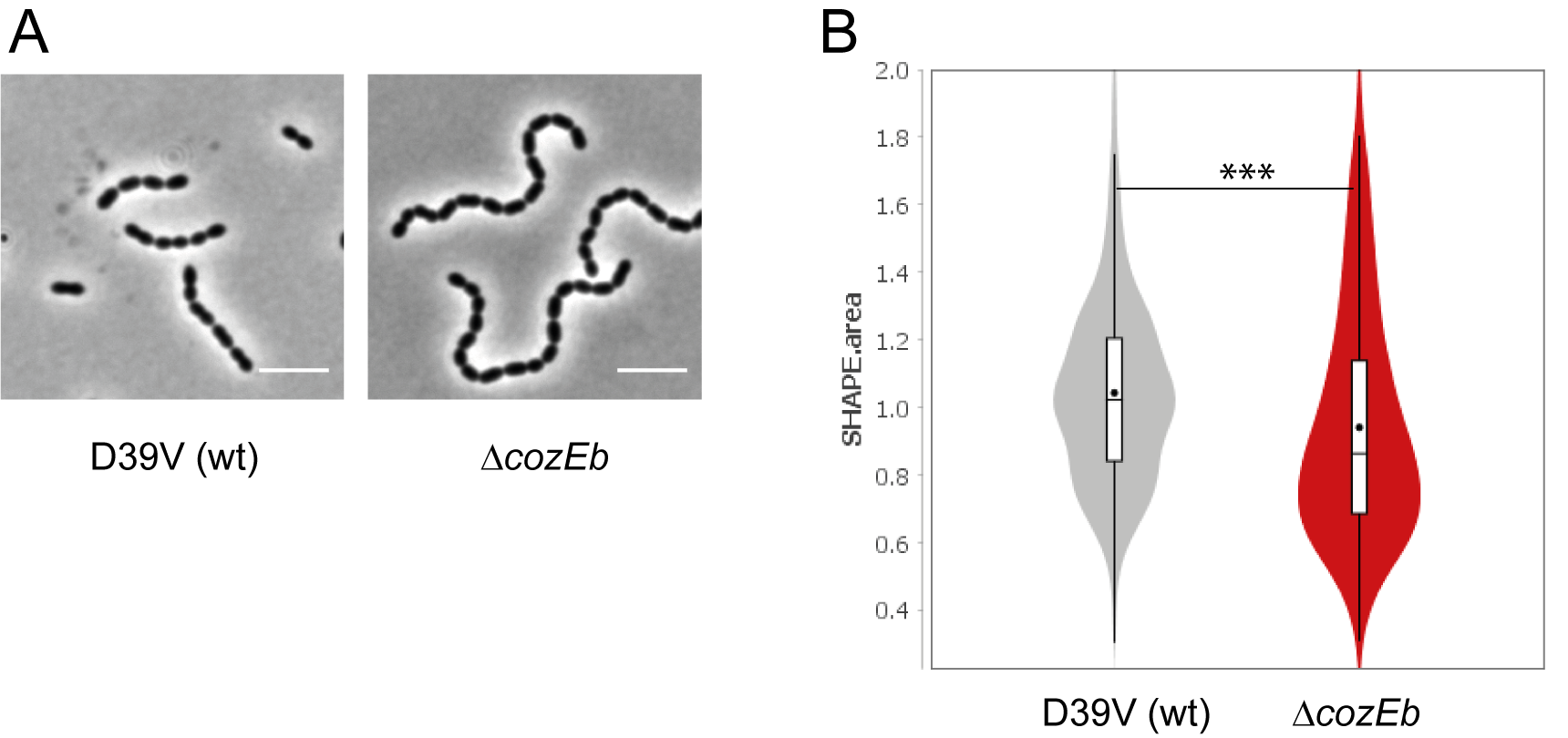

Supplement: FIG S3 [file mBio.02461-20-sf003.tif]

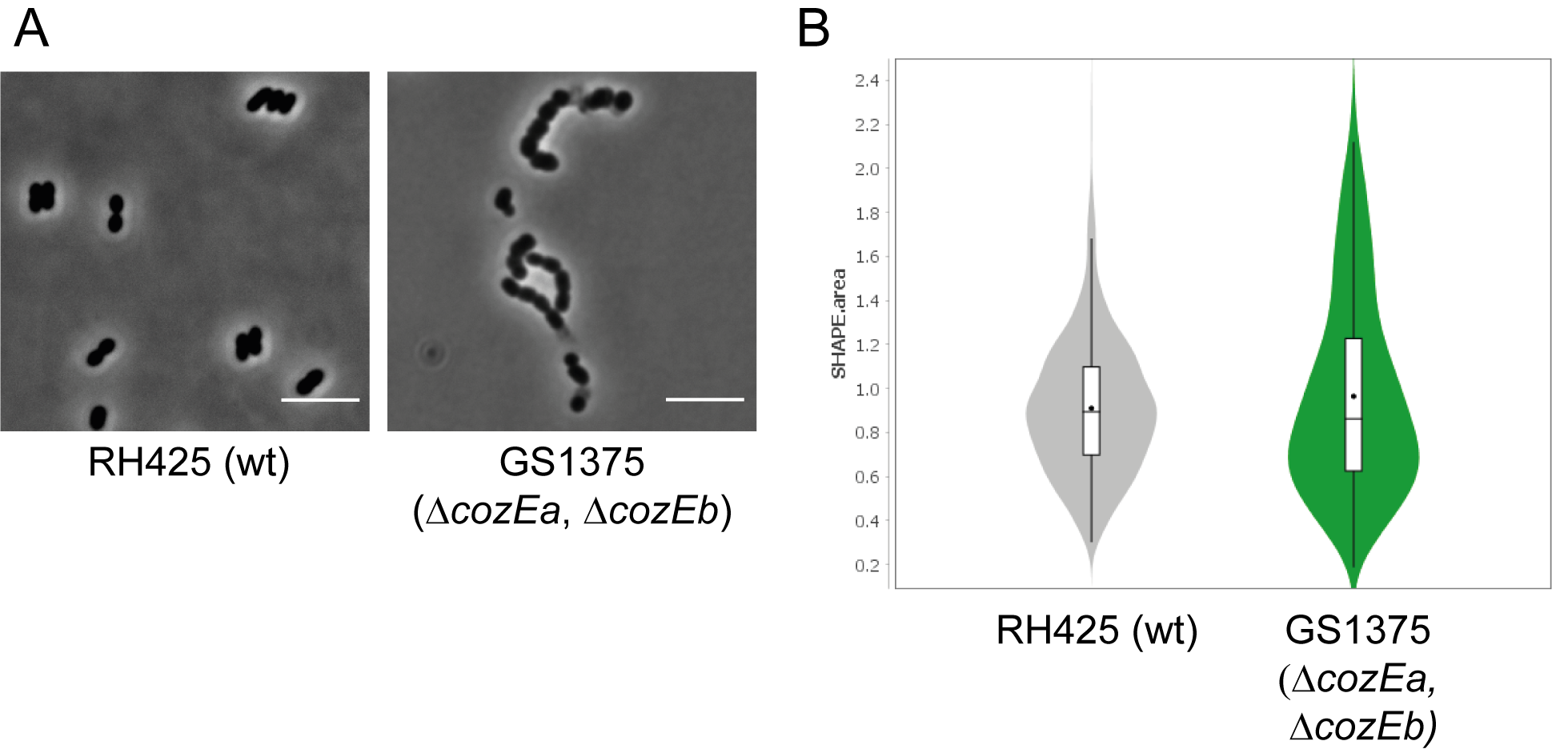

Supplement: FIG S4 [file mBio.02461-20-sf004.tif]

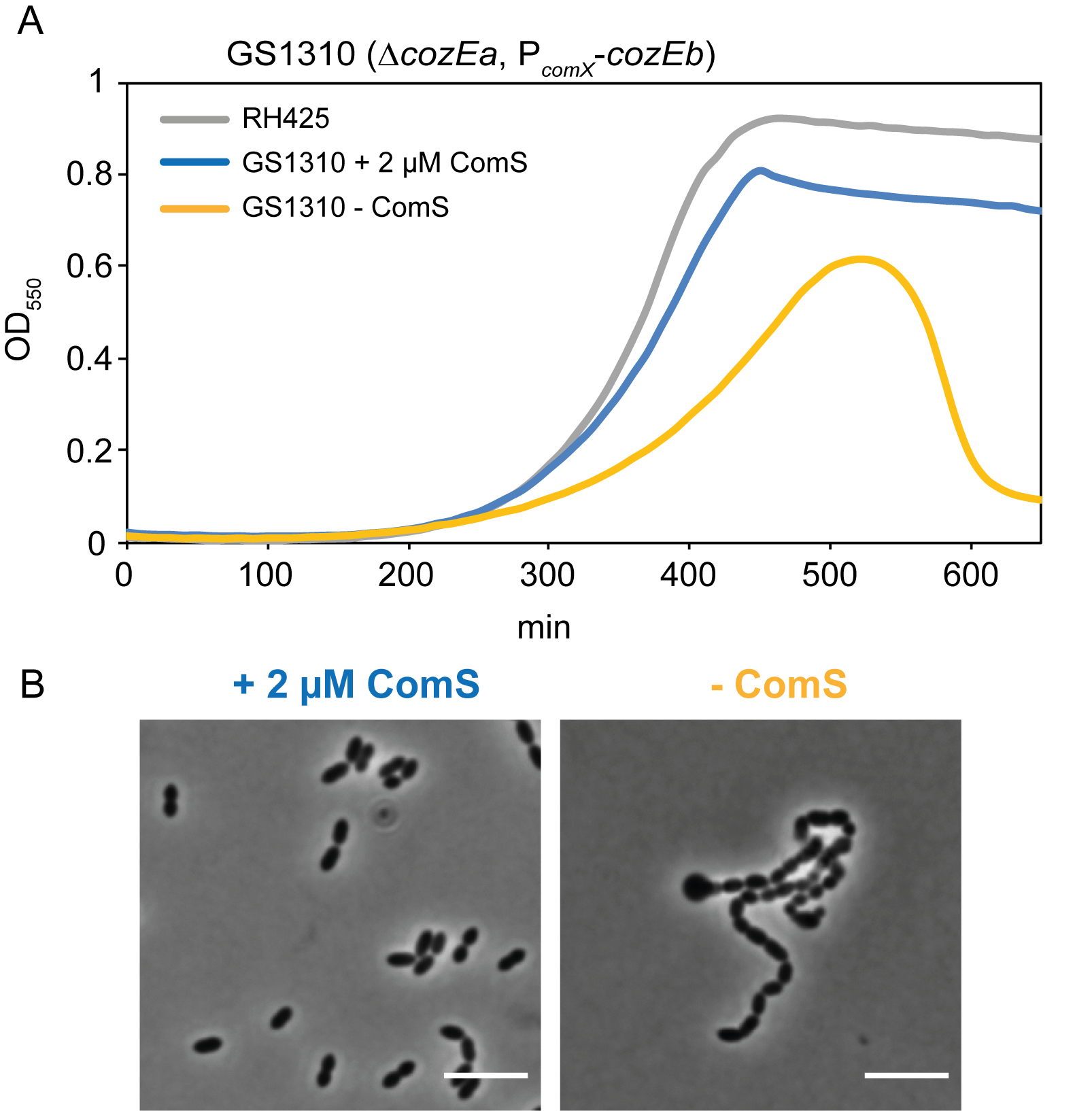

Supplement: FIG S5 [file mBio.02461-20-sf005.tif]

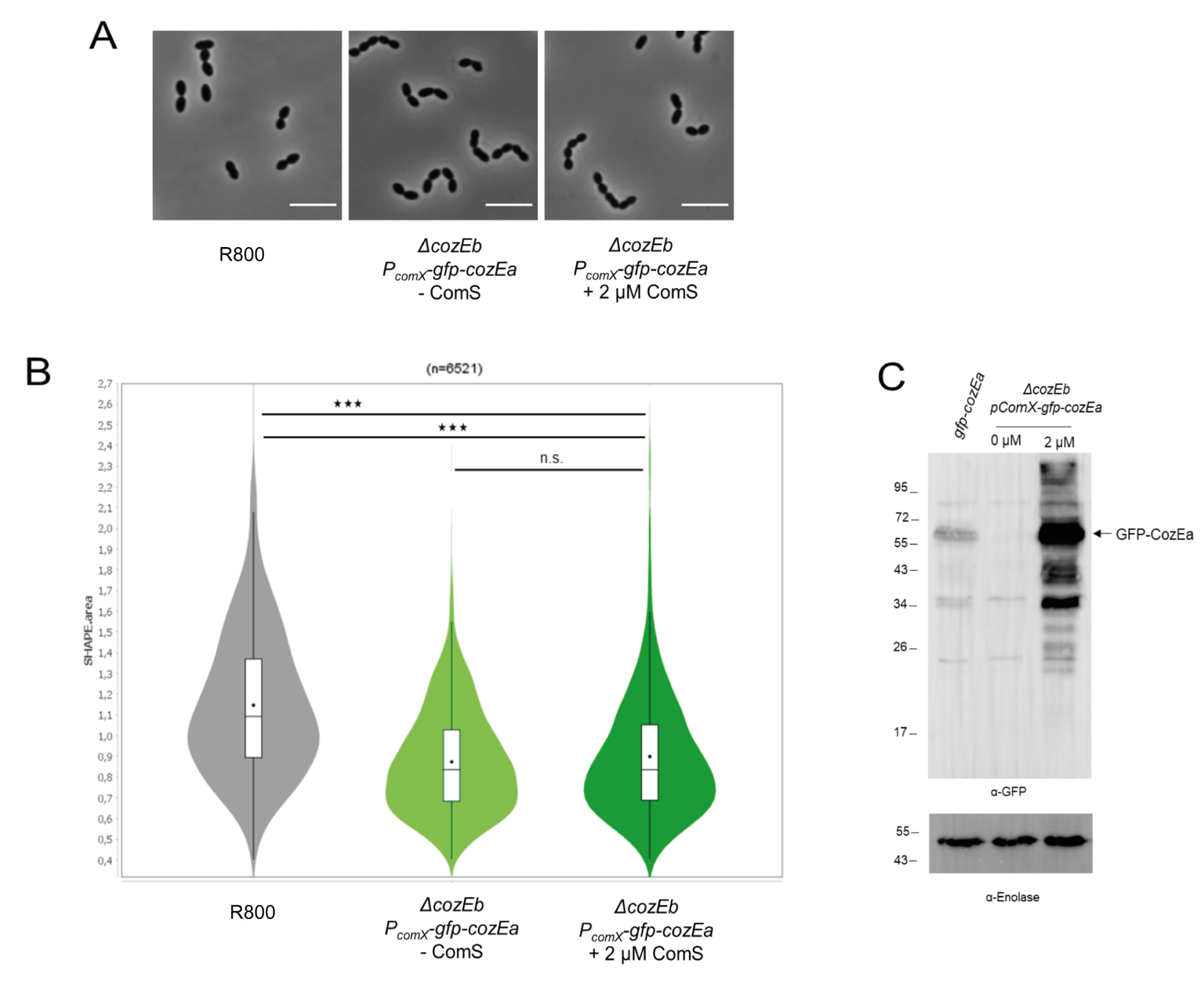

Supplement: FIG S6 [file mBio.02461-20-sf006.tif]

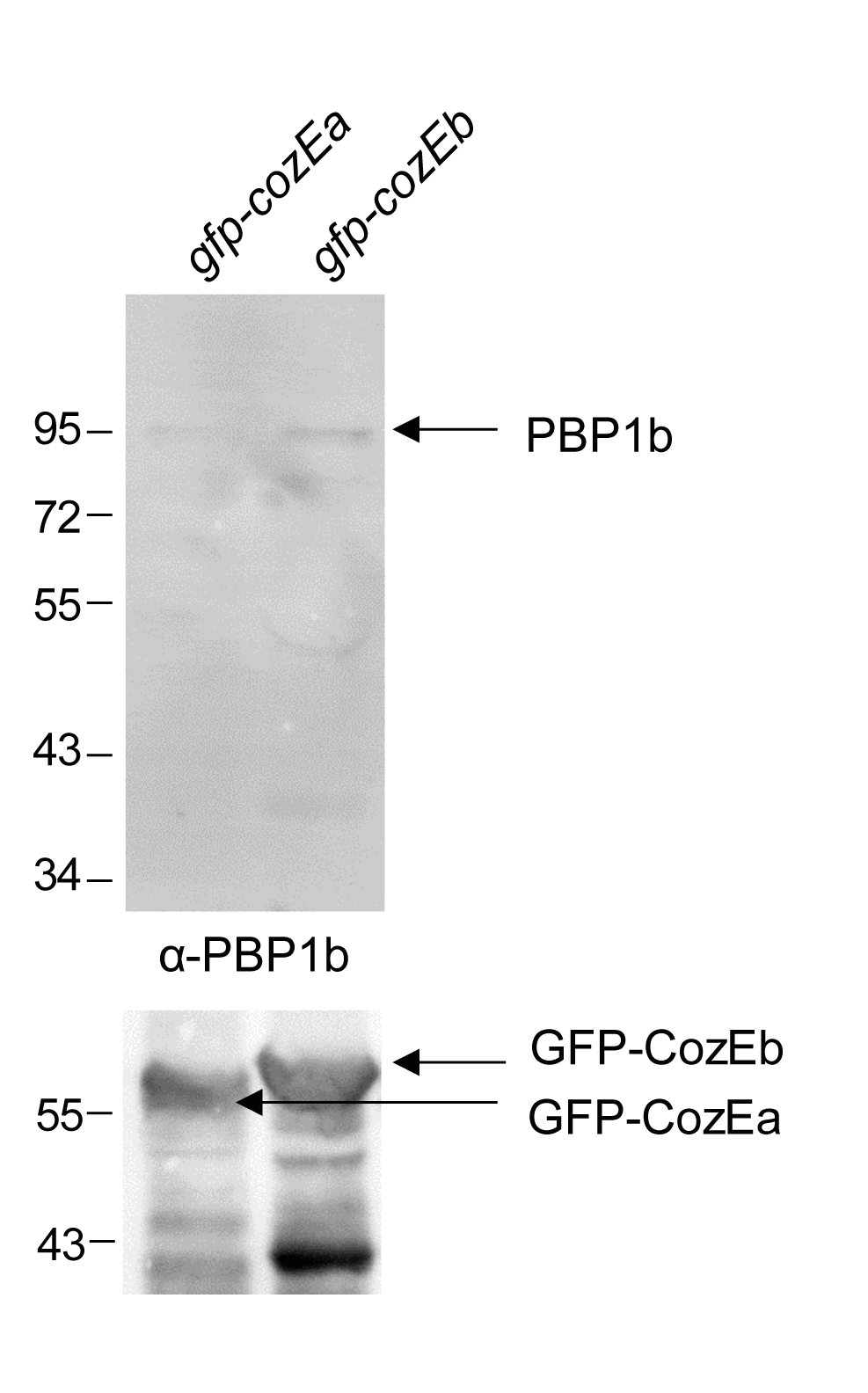

Supplement: FIG S7 [file mBio.02461-20-sf007.tif]
