# Supplementary material for: A CozE Homolog Contributes to Cell Size Homeostasis of Streptococcus pneumoniae
Source: mBio. 2020 Oct 27;11(5):e02461-20. doi: 10.1128/mBio.02461-20 (PMC7593971; doi:10.1128/mBio.02461-20)
Supplement: TABLE S1 [file mBio.02461-20-st001.pdf]

Table S1. Plasmids and strains used in this study

Strain name	Genotype; antibiotic resistance[a]	References	Primers[b]
RH425 (WT)	Δ*comA*::*ery*, *rpsL*::*rpsL1*; Sm^R	(1)	
D39V (WT)		(2)	
R800 (WT)	R800 (R6 derivative) *rpsL*::*rpsL1*; Sm^R	(3)	
SPH154	RH425, Δ*IS1167*-P1-P$_{comR}$::*comR*, *cpsO-cpsN*::Janus, Kan^R	(4)	
SPH131	RH425, Δ*IS1167*-P1-P$_{comR}$::*comR*, *cpsO-cpsN::P$_{comX}$*-Janus; Kan^R	(4)	
SPH355	RH425, *cpsO-cpsN*::P$_{comX}$-*cozEa*, Δ*cozEa*; Sm^R	(5)	
GS870	RH425: Δ*cozEb*::Janus; Kan^R	This work	KHB498-KHB499; KHB500-KHB501; Kan484.F-RpsL41.R
GS1101	RH425, Δ*cozEa*::Janus; Kan^R	This work	KHB482-KHB483; KHB484-KHB485; Kan484.F-RpsL41.R
GS1250	RH425, Δ*cozEb*; Sm^R	This work	KHB498-GS722; GS723-KHB501
GS1279	RH425, *cpsO-cpsN*::P$_{comX}$ -*cozEb*; Sm^R	This work	KHB31-KHB36; KHB33-KHB34; GS730-GS731
GS1310	RH425, *cpsO-cpsN*::P$_{comX}$ -*cozEb*, Δ*cozEa*::Janus; Kan^R	This work	KHB482-KHB483; KHB484-KHB485; Kan484.F-RpsL41.R
GS1336	D39V, Δ*cozEb*::Janus; Kan^R	This work	KHB498-KHB499; KHB500-KHB501; Kan484.F-RpsL41.R
GS1375	RH425, Δ*cozEa*, Δ*cozEb*::Janus; Kan^R	This work	KHB498-KHB499; KHB500-KHB501; Kan484.F-RpsL41.R
Spn168	R800 *rpsL*::*rpsL1*; Δ*bgaA::P$_{Zn}$::gfp-pbp1a*; Sm^R, Tet^R	(6)	
Spn1010	R800 *rpsL*::*rpsL1*, Δ*IS1167*::P1::P$_{comR}$::*comR*, *cpsO-cpsN::P$_{comX}$*-Janus; Kan^R	(7)	
Spn1496	R800 *rpsL*::*rpsL1*, *ΔcozEb*::Janus; Kan^R	This work	2639-2642; 2663-2640; 536-537
Spn1497	R800 *rpsL*::*rpsL1*, *ΔcozEb*; Sm^R	This work	2639-2665; 2664-2640
Spn1528	R800 *rpsL*::*rpsL1*, *gfp-cozEb*; Sm^R	This work	2639-2689; 2688-2640; 1368-1568
Spn1680	R800 *rpsL*::*rpsL1*, Δ*cozEa*::Janus; Kan^R	This work	2789-2660; 2659-2790; 536-537
Spn1681	R800 *rpsL*::*rpsL1*, Δ*cozEa*; Sm^R	This work	2789-2662; 2661-2790
Spn1682	R800 *rpsL*::*rpsL1*, *gfp-cozEa*; Sm^R	This work	2789-2691; 2817-2790; 2216-2818
Spn1884	R800 *rpsL*::*rpsL1*; Δ*IS1167*::P1::P$_{comR}$::*comR*, *cpsO-cpsN::P$_{comX}$-gfp-cozEa*; Sm^R	This work	1943-2082; 2672-2746 ; 1946-2081
Spn1885	R800 *rpsL*::*rpsL1*; Δ*IS1167*::P1::P$_{comR}$::*comR*, *cpsO-cpsN::P$_{comX}$-gfp-cozEb*; Sm^R	This work	1943-2082; 2670-2746; 1946-2081
Spn1922	R800 *rpsL*::*rpsL1*, *gfp-cozEb*; Δ*cozEa*::Janus; Kan^R	This work	2789-2660; 2659-2790; 536-537
Spn1928	R800 *rpsL*::*rpsL1*, *gfp-cozEb*, *flag-cozEa*; Sm^R	This work	2789-2996; 2820-2790

Spn1955	R800 *rpsL*::*rpsL1*; Δ*cozEa*::Janus; Δ*IS1167*::P1-P_{comR}-*comR*, *cpsO-cpsN::*P_{comX}-*gfp-cozEb*; Kan^R	This work	2789-2660; 2659-2790; 536-537
Spn1945	R800 *rpsL*::*rpsL1*; Δ*cozEa*; Δ*IS1167*::P1::P_{comR}::*comR*, *cpsO-cpsN::*P_{comX}-*gfp-cozEb*; Sm^R	This work	2789-2662; 2661-2790
Spn1992	R800 *rpsL*::*rpsL1;* Δ*cozEa::*janus; *ΔbgaA::Pzn::gfp-pbp1a*; KanR, Tet^R	This work	2789-2660; 2659-2790; 536-537
Spn1993	R800 *rpsL*::*rpsL1*; Δ*cozEa; ΔbgaA::Pzn::gfp-pbp1a*; Sm^R, Tet^R	This work	2789-2662; 2661-2790
Spn1994	R800 *rpsL*::*rpsL1*; Δ*cozEb*::janus; *ΔbgaA::Pzn::gfp-pbp1a*; Kan^R, Tet^R	This work	2639-2642; 2663-2640; 536-537
Spn1995	R800 *rpsL*::*rpsL1*; Δ*cozEb; ΔbgaA::Pzn::gfp-pbp1a*; Sm^R, Tet^R	This work	2639-2665; 2664-2640
Spn2016	R800 *rpsL*::*rpsL1*; Δ*cozEa*; Δ*IS1167*::P1::P_{comR}::*comR*; Sm^R	This work	946-947
Spn2017	R800 *rpsL*::*rpsL1*; Δ*cozEa*; Δ*IS1167*::P1::P_{comR}::*comR*, *cpsO-cpsN::*P_{comX}-janus; Kan^R	This work	1943-1946
Spn2018	R800 *rpsL*::*rpsL1*; Δ*cozEa*; Δ*IS1167*::P1::P_{comR}::*comR*, *cpsO-cpsN::*P_{comX}-*cozEb*; Sm^R	This work	1943-2082; 2669-2670; 1946-2081
Spn2026	R800 *rpsL*::*rpsL1*, Δ*cozEa*::*spc*; Sm^R, Spc^R	(8), This work	2789-2790
Spn2027	R800 *rpsL*::*rpsL1*, Δ*cozEb*; Δ*cozEa*::*spc*; Sm^R, Spc^R	This work	2789-2790
Spn2031	R800 *rpsL*::*rpsL1*; Δ*cozEa*; Δ*bgaA*::P_{Zn}-*gfp-pbp1a*, Δ*IS1167*::P1-P_{comR}-*comR*, *cpsO-cpsN::*P_{comX}-*cozEb*; Sm^R, Tet^R	This work	

[a] Sm^R, streptomycin resistant; Kan^R, kanamycin resistant; Tet^R, tetracycline resistant, Spc^R, spectinomycin resistant

[b] Primers sequences are found in Table S2.

References

1. Johnsborg O, Håvarstein LS. 2009. Pneumococcal LytR, a protein from the LytR-CpsA-Psr family, is essential for normal septum formation in *Streptococcus pneumoniae*. J Bacteriol 191:5859-64.
2. Slager J, Aprianto R, Veening J-W. 2018. Deep genome annotation of the opportunistic human pathogen *Streptococcus pneumoniae* D39. Nucleic Acids Res 46:9971-9989.
3. Fleurie A, Cluzel C, Guiral S, Freton C, Galisson F, Zanella-Cleon I, Di Guilmi AM, Grangeasse C. 2012. Mutational dissection of the S/T-kinase StkP reveals crucial roles in cell division of *Streptococcus pneumoniae*. Mol Microbiol 83:746-58.
4. Berg KH, Biørnstad TJ, Straume D, Håvarstein LS. 2011. Peptide-regulated gene depletion system developed for use in *Streptococcus pneumoniae*. J Bacteriol 193:5207-15.
5. Straume D, Stamsås GA, Berg KH, Salehian Z, Håvarstein LS. 2017. Identification of pneumococcal proteins that are functionally linked to penicillin-binding protein 2b (PBP2b). Mol Microbiol 103:99-116.

6. Peters K, Schweizer I, Beilharz K, Stahlmann C, Veening JW, Hakenbeck R, Denapaite D. 2014. *Streptococcus pneumoniae* PBP2x mid-cell localization requires the C-terminal PASTA domains and is essential for cell shape maintenance. Mol Microbiol 92:733-55.
7. Zucchini L, Mercy C, Garcia PS, Cluzel C, Gueguen-Chaignon V, Galisson F, Freton C, Guiral S, Brochier-Armanet C, Gouet P, Grangeasse C. 2018. PASTA repeats of the protein kinase StkP interconnect cell constriction and separation of *Streptococcus pneumoniae*. Nat Microbiol 3:197-209.
8. Fenton AK, Mortaji LE, Lau DT, Rudner DZ, Bernhardt TG. 2016. CozE is a member of the MreCD complex that directs cell elongation in *Streptococcus pneumoniae*. Nat Microbiol 2:16237.
