# Supplementary material for: A CozE Homolog Contributes to Cell Size Homeostasis of Streptococcus pneumoniae
Source: mBio. 2020 Oct 27;11(5):e02461-20. doi: 10.1128/mBio.02461-20 (PMC7593971; doi:10.1128/mBio.02461-20)
Supplement: TABLE S2 [file mBio.02461-20-st002.pdf]

Table S2. List of oligos used in this study

Name	Sequence (5'-3')
Kan484.F	GTTTGATTTTTAATGGATAATGTG
RpsL41.R	CTTTCCTTATGCTTTTGGAC
GS473	GAGTTACCTCCCTCACTTTAT
GS474	ATAAAGTGAGGGAGGTAACTCAAGTCAGGAGAACCCTGATTT
GS722	AAATACTTCCTTTCTATTGTTCTC
GS723	GAGAACAATAGAAAGGAAGTATTTGGTAGTCAGTGGTCTATATGAAT
GS730	GAATTTATATTTATTATTGGAGGTTCAATGGAGCAAAAAGAGAAACATTTTAG
GS731	ATTGGGAAGAGTTACATATTAGAAACTATTGTTCACTCTTGACTTCC
GS747	GAGCTTCAGATGTACGATTATCCATTACTATACCTCTGTGTAAAG
GS748	TATGTTTTTGGCGGATCTCATAAATACTTCCTTTCTATTGTTCTC
GS749	GAGCTTCAGATGTACGATTATCGAGCAACAGCAGTAGAAACAC
GS750	TATGTTTTTGGCGGATCTCATGAGTTACCTCCCTCACTTTATTTTAC
GS91	CTAGCGGTTCGTAGTTTACCAATTACAATTTGGGCTTTCCGC
KHB31	ATAACAAATCCAGTAGCTTTGG
KHB33	TTTCTAATATGTAACTCTTCCCAAT
KHB34	CATCGGAACCTATACTCTTTTAG
KHB36	TGAACCTCCAATAATAAATATAAAT
KHB482	ACGATTTTGCGAAGTGTAAATG
KHB483	CACATTATCCATTAAAAATCAAACGAGTTACCTCCCTCACTTTAT
KHB484	GTCCAAAAGCATAAGGAAAGAAGTCAGGAGAACCCTGATTT
KHB485	AAGGAATAATGGAGCCGGTG
KHB497	ATTGGGAAGAGTTACATATTAGAAACTATTGTTCACTCTTGACTTCC
KHB498	TCACGTGGAGTCTGACCATG
KHB499	CACATTATCCATTAAAAATCAAACAAATACTTCCTTTCTATTGTTCTC
KHB50	CCGATGCAGAAATGGTTGAG
KHB500	GTCCAAAAGCATAAGGAAAGGTAGTCAGTGGTCTATATGAAT
KHB501	CTGGCTCCTCACTCTGCAA
KHB51	GATAATCGTACATCTGAAGCTC
KHB52	TTGGTAAACTACGAACCGCTAG
KHB78	ATGAGATCCGCCAAAAACATA
2639	GGTGATGACTTGGTCAGTTCCC
2640	GCCTGACTGTACCCATACTC
2665	CCTCACCCTCTAATTCATATAGACCAAAATACTTCCTTTCTATTGTTCTCTC
2664	GAGAGAACAATAGAAAGGAAGTATTTTGGTCTATATGAATTAGAGGGTGAGG
2642	CATTATCCATTAAAAATCAAACGGAAATACTTCCTTTCTATTGTTCTCTC
2663	GGAAAGGGGCCCAGGTCTCTTGGTCTATATGAATTAGAGGGTGAGG
536	CCGTTTGATTTTTAATGGATAATG
537	AGAGACCTGGGCCCCTTTCC
2688	GTATAAACTCGAGGGATCCGGAATGGAGCAAAAAGAGAAACATTTTAGCC
2689	AATTCTTCACCTTTAGAAATCATAAATACTTCCTTTCTATTGTTCTCTC
2688	GTATAAACTCGAGGGATCCGGAATGGAGCAAAAAGAGAAACATTTTAGCC
2689	AATTCTTCACCTTTAGAAATCATAAATACTTCCTTTCTATTGTTCTCTC

1368	ATGATTTCTAAAGGTGAAGAATTG
1568	TCCGGATCCCTCGAG TTTATACAATTCATCCATACCATGTG
2789	CCTGGGTAAATGTTATCCCC
2790	CGAATCCGTAACGACCCCAAAAG
2660	CATTATCCATTAAAAATCAAACGGGAGTTACCTCCCTCACTTTATTTTACC
2659	GGAAAGGGGCCCAGGTCTCTAAGTCAGGAGAACCCTGATTTTTCTTTACTGG
2661	GGTAAAATAAAGTGAGGGAGGTAACTCAAGTCAGGAGAACCCTGATTTTTCTTTAC TGG
2662	CCAGTAAAGAAAAATCAGGGTTCTCCTGACTTGAGTTACCTCCCTCACTTTATTTTA CC
2691	AATTCTTCACCTTTAGAAATCAT GAGTTACCTCCCTCACTTTATTTTACC
2817	GAACGAGAAAGAGAATTAGCTAAGTAAAAGTCAGGAGAACCCTGATTTTTCTTTA CTGG
2216	ATGATTTCTAAAGGTGAAGAATT
2818	CCAGTAAAGAAAAATCAGGGTTCTCCTGACTTTTACTTAGCTAATTCTCTTTCTCGT TC
2996	CGATATCATGATCTTTATAATCACCGTCATGGTCTTTGTAGTCCATGAGTTACCTCC CTCACTTTATTTTACC
2820	ATGGACTACAAAGACCATGACGGTGATTATAAAGATCATGATATCGACTACAAAG ATGACGACGATAAACTCGAGGGATCCGGAATGTTTCGTAGAAATAAATTATTTTT TGGACCAC
1943	CATCGGAACCTATACTCTTTTAG
1946	ATAACAAATCCAGTAGCTTTGG
2082	TTTCTAATATGTAACTCTTCCCAAT
2672	ATTGGGAAGAGTTACATATTAGAAATTACTTAGCTAATTCTCTTTCTCGTTC
2081	TGAACCTCCAATAATAAATATAAAT
2670	ATTGGGAAGAGTTACATATTAGAAACTATTGTTCACTCTTGACTTCCTCACCC
2746	ATTTATATTTATTATTGGAGGTTCAATGATTTCTAAAGGTGAAGAATTG
946	ATGAAGAAATACCAACAATTATTTAAGCAAATCC
947	ACCCTCAT GAATTC TCAGGCGGT
2669	ATTTATATTTATTATTGGAGGTTCAATGGAGCAAAAAGAGAAACATTTTAGCCTAT CTTGG
